# COVID-19 exit strategy during vaccine implementation: a balance between social distancing and herd immunity

**DOI:** 10.1007/s00705-022-05495-7

**Published:** 2022-06-20

**Authors:** Suhad Daher-Nashif, Rania Al-Anany, Menatalla Ali, Khadija Erradi, Elmoubasher Farag, Abdallah M. Abdallah, Mohamed M. Emara

**Affiliations:** 1 Population Medicine Department, College of Medicine, QU Health, Qatar University, Doha, Qatar; 2 Basic Medical Sciences Department, College of Medicine, QU Health, Qatar University, Doha, Qatar; 3 Public Health Department, Health Protection and Communicable Diseases, Ministry of Public Health, Doha, Qatar

## Abstract

Currently, health authorities around the world are struggling to limit the spread of COVID-19. Since the beginning of the pandemic, social distancing has been the most important strategy used by most countries to control disease spread by flattening and elongating the epidemic curve. Another strategy, herd immunity, was also applied by some countries through relaxed control measures that allow the free spread of natural infection to build up solid immunity within the population. In 2021, COVID-19 vaccination was introduced with tremendous effort as a promising strategy for limiting the spread of disease. Therefore, in this review, we present the current knowledge about social distancing, herd immunity strategies, and aspects of their implementation to control the COVID-19 pandemic in the presence of the newly developed vaccines. Finally, we suggest a short-term option for controlling the pandemic during vaccine application.

## Introduction

Coronavirus disease 19 (COVID-19) first emerged in Wuhan in December 2019 and since then has been expanding rapidly all over the globe [1–3]. By October 8, 2021, over 236 million confirmed cases and more than 4.8 million deaths had been recorded [4]. As the number of fatalities increased [1], so did the pressure and urgency to develop safe and effective vaccines in order to control the pandemic. In fact, by October 8, 2021, up to 194 vaccine candidates were in the preclinical development phase and 126 vaccines were already in the clinical trial phase [5]. Because of the pandemic, the US Food and Drug Administration (FDA) issued an Emergency Use Authorization for the development of vaccines [6], and it approved the distribution of the first COVID-19 vaccines in the US as early as December 2020 [7]. Initially, this decision raised some concerns regarding whether rigorous safety and efficacy testing had been compromised for the sake of urgent development [8, 9]. Fortunately, however, it has been confirmed that the vaccines that were produced are safe and effective [10–13]. Without a doubt, the progress in vaccine development that was made in such a short time while maintaining the required safety and efficacy standards is an astounding achievement [14]. It is important to note, however, that development of vaccines does not guarantee their availability, accessibility, or efficacy for everyone [15, 16]. For example, it has been estimated that vaccines would not be accessible for 90% of people in lower-income countries until 2023 [15, 17]. In addition, anti-vaccination movements and hesitancy across the world pose a risk that herd immunity will not be achieved [15, 18, 19]. Initially, a lot of uncertainty surrounded the vaccines and their development, including reinfections and the possibility of resistance due to the high genetic variability of SARS-CoV-2 [18, 20]. It is also important to note that vaccination might not reduce SARS-CoV-2 transmission as efficiently as it can protect individuals from the disease [21]. This could easily become a double-edged sword, since vaccinated individuals may think they are safe and socialize freely while carrying the virus asymptomatically and spreading the infection [22].

For these reasons, it is essential to implement parallel strategies to manage the pandemic [15]. Most of these strategies aim to (i) protect individuals at high risk of severe illness or death, (ii) reduce the overall number of cases, thereby reducing peak morbidity and mortality, (iii) decrease stress on healthcare services and infrastructure, and (iv) buy time for the development of antiviral therapies and increase the global accessibility of vaccines. Of these strategies, social distancing and herd immunity stand out as the two most prominent strategies currently applied to control COVID-19. The social distancing strategy focuses on reducing transmission with the goal of flattening and elongating the epidemic curve [23], whereas herd immunity through natural infection and/or vaccination is applied to build up solid immunity within a specific population [24]. The former strategy will help minimize COVID-19 transmission levels, while the latter can aid vaccines in reaching threshold herd immunity levels through natural and/or artificial infections. Therefore, in this review, we present the current knowledge about social distancing and herd immunity strategies and the aspects of their implementation to control the COVID-19 pandemic. We also discuss COVID-19 vaccination, its relationship to social distancing, and the challenges associated with vaccine implementation. Finally, we suggest a short-term option for controlling the pandemic during vaccine application.

## COVID-19 and social distancing

Social distancing is a set of practices undertaken to increase the physical distance between people and reduce the frequency of congregation, with the goal of minimizing the spread of an infectious disease [23]. Community transmission of SARS-CoV-2 is occurring globally, leading to large numbers of undetected infected individuals who could infect others [25]. To limit SARS-CoV-2 transmission, social distancing measures have been implemented in most countries around the world [26]. This strategy was also used during the SARS outbreak in 2003, when Singapore implemented many physical distancing measures, including “wide-net” surveillance, isolation, and quarantine of potential SARS cases, and successfully contained the outbreak [27]. Similar measures were enforced in other affected countries, leading to the suppression of all human-to-human transmission of SARS [28].

The application of mathematical modeling that simulates the trajectory of outbreaks and helps in understanding the effects of physical distancing confirms the importance of social distancing in controlling the outbreak. For instance, modeling studies have shown that workplace social distancing measures alone can reduce transmission rates of influenza and delay the peak of the infection [29]. Furthermore, one study that modeled the COVID-19 outbreak in Wuhan estimated that enforcing physical distancing measures until early April would reduce the median number of infections by more than 92% by the middle of 2020, and by 24% by the end of 2020 [30]. Another study estimated that if social distancing measures were to be relaxed by 2% in South Africa, the number of cumulative COVID-19 cases could increase by 23% [31]. This is contrary to an 18% decrease in the number of cumulative COVID-19 cases if social distancing measures were to be increased only by 2% [31]. This model was deemed accurate, as it correctly predicted the number or cases after South Africa relaxed social distancing measures. Similarly, a model predicted that if social distancing measures were completely lifted in four US states, 0.8–4 million new COVID-19 cases and 15,000–240,000 deaths would occur over a year [32]. If social distancing and other protective measures such as wearing medical masks were to be removed, a model predicted that a vaccine with 100% efficacy will have to be implemented and have a coverage of 33-58% in order to overcome the pandemic [32]. Indeed, social distancing strategies have been associated with significant reductions in the effective reproduction number (R_t_) across the globe [33–35]. Importantly, implementation of social distancing on a large scale requires the intervention of law enforcement and may include legal penalties for violators [36]. The application of such legal intervention may increase compliance with social distancing [37] and help reduce the number of infections [38–40].

Although social distancing is a very effective measure for controlling virus spread, it places significant stress on the economy, leading to reduced income and unemployment [41–43]. Certainly, COVID-19 and the social distancing measures taken in response to it have revealed inequalities due to many factors [44], including sex [45], race [46–48], technological advances and access [49–51], and poverty [17]. One specific disadvantage of social distancing is the loss of social connectedness amongst people, which has been correlated to morbidity. This issue has been referred to as the “COVID-19 connectivity paradox” [52]. This refers to the conflict between the perceived benefit of social distancing measures and the harm inflicted upon vulnerable populations such as the elderly due to isolation and lack of connectedness, leading to both mental and physical illnesses [52]. This phenomenon was demonstrated and reported in Italy, where the number of COVID-19 infections in elderly individuals (>80 years) was higher in areas with increased family separation and in areas with a higher level of nursing home availability [53]. Another report demonstrated a significant association between social isolation and mortality, with the increased death rate in adults reaching up to 35% compared to those receiving social support, even after excluding important disease risk factors [54]. Moreover, the lack of social connectedness has negatively affected vulnerable groups, such as individuals with chronic illnesses (especially cancer) and those without internet or technology access [55], more than the rest of the population.

Therefore, policymakers should ensure that their social distancing measures are implemented for as long as necessary and that they do not discriminate against any population group or take actions leading to social injustice. Due to the severe economic impact of social distancing, countries have started to relax quarantine measures gradually [2]. If done prematurely and suddenly, this can lead to a second surge in infections [56]. A study showed that after the US relaxed social distancing measures, virus transmission increased eight weeks later [57]. Therefore, population-wide social distancing should be maintained until populations are immunized via vaccines, but this needs to be done in a way that helps people to withstand the negative economic impact of social distancing [15, 58].

## COVID-19 and herd immunity

Herd immunity occurs when a large proportion of the population has become immune to an infectious agent, thereby stopping or slowing its transmission [24]. Indirectly, it shields susceptible individuals in the population from becoming infected*.* Herd immunity can be achieved either through natural infection or vaccination. Herd immunity through natural infection allows the virus to spread freely. This was the initial strategy of the UK government and is the current strategy in Sweden [59, 60]. However, allowing the virus to spread freely can spiral out of control and have far-reaching consequences. One study has estimated that if infections were left unchecked, there would be approximately 510,000 deaths in Great Britain and 2.2 million in the United States [61]. The high levels of morbidity can rapidly overwhelm healthcare systems and worsen outcomes for critically ill patients, especially in countries with weak healthcare systems and low socio-economic status [62]. It is important to note, however, that the use of herd immunity through natural infection as a strategy to suppress SARS-CoV-2, especially in areas where vaccinations are not available or accessible, assumes that individuals that recover from the infection will have adequate protective immunity [63].

One way of assessing immunity has been by testing for the presence of neutralizing antibodies [64]. Several countries, such as the USA, Chile, and Germany, have considered the idea of providing “immunity certificates” to recovered COVID-19 patients who test positive for antibodies to SARS-CoV-2 [65]. Neutralizing antibodies can provide protective immunity and prevent future viral reinfection by preventing the virus from entering cells [66]. In contrast, a nationwide serological survey conducted in the Netherlands to estimate the prevalence of SARS-CoV-2 antibodies reported that the level of immunity in the Netherlands is below the required threshold to achieve herd immunity and concluded that natural herd immunity is not a viable short-term strategy for allowing social distancing measures to be lifted [67]. Accordingly, although it has been estimated that more than 75% of the population in Manaus, Brazil, has been infected with SARS-CoV-2 [68], which is a level that would normally be sufficient to achieve herd immunity, COVID-19 has resurged [68]. This could have been due to an overestimate of the number of infections, in which case the necessary number of infected individuals for reaching herd immunity had not actually been reached. Another explanation could be the fact that SARS-CoV-2 variants were able to evade immunity [69] and also became even more infectious. Altogether, it can be argued that herd immunity alone, whether natural or through vaccination, cannot guarantee disease eradication, making it necessary to implement additional protective measures and strategies [15, 67].

## COVID-19 vaccination and its challenges

COVID-19 vaccination is considered one of the strategies that will help to limit the spread of infection. Accordingly, several companies are working on developing safe and effective vaccines [70]. Generally, COVID-19 candidate vaccines can be classified based on the vaccine composition: (i) inactivated virus, which is not capable of replication but still can trigger an immune response [71–73]; (ii) live-attenuated virus, which has weak pathogenicity or none at all, while maintaining its capacity to trigger the immune system [74]; (iii) protein subunits, consisting only of the major viral proteins, the class into which most COVID-19 vaccines fall [74–77]; (iv) virus-vectored, where immunogenic antigens are produced by cloning their genes into virus vectors [71, 78, 79]; and (v) DNA and (vi) mRNA vaccines, in which viral antigens encoded by recombinant DNA [80] or mRNA are produced by protein translation in the host cell [81–83].

These candidate vaccines are designed to target the four structural proteins composing the SARS-CoV-2 virion: (i) the spike (S) protein, which mediates binding, entry, and attachment to host cells, (ii) the membrane (M) protein, a component of the envelope of the virion, (iii) the envelope (E) protein, which is required for infection, and (iv) the nucleocapsid (N) protein, which forms the ribonucleoprotein core [84]. Although these four proteins act as potential targets for COVID-19 vaccine development, the spike protein is the main target of the current vaccines [85] because neutralizing antibodies mainly target this protein [85]. However, the SARS-CoV-2 spike protein has undergone numerous mutations in different strains, which raises the question of whether the virus will eventually mutate to an extent that it will become resistant to vaccines [86, 87]. Although numerous clinical trials have provided evidence of the safety and efficacy of most COVID-19 vaccines [88–90], these studies are limited by the fact that they cannot provide long-term evidence, as well as the fact that their sample populations tend to consist exclusively of adolescents and diseased individuals [12]. Furthermore, a systemic review has shown that evidence regarding the appropriate interval between the two doses recommended for most COVID-19 vaccines is still lacking and that further study is needed [12]. Another aspect to note is that the study design and protocol differ amongst these trials, thus complicating comparisons between the results and making it difficult to replicate these studies using different population demographics [12].

The emergence of new SARS-CoV-2 strains remains one of the main challenges to successful implementation of vaccines, requiring their efficacy and safety to be reassessed [91, 92]. Generally, RNA viruses such as coronaviruses have high rates of mutation, leading to changes in virulence factors associated with disease severity [93, 94]. The coronavirus genome is highly susceptible to genetic mutations that can lead to immune evasion [95]. Some of the mutations in the SARS-CoV-2 genome have been shown to influence transmission, pathogenicity, and immunity [94].

Although mutations have not been shown to affect vaccine efficacy [96], the continuing mutation of SARS-CoV-2 raises doubts regarding vaccine efficacy in the future [87, 97, 98]. For instance, a SARS-CoV-2 strain that emerged in Europe in early 2020 has been identified to have a D614G mutation in the spike protein [99]. This mutation led to an increase in levels of transmission and viral loads compared to the initial D614 strain. Another variant known as the B.1.1.7 or Alpha variant, which emerged in the UK in December 2020, also included a substitution (N501Y) and a deletion (69-70del) in the spike protein that strengthened its interaction with the ACE2 receptor, which is used by the virus for cell entry [100], leading to high transmissibility, specifically an increase of 60% compared to the original SARS-CoV-2 strain [101]. Another strain that emerged in South Africa, known as the Beta or B.1.351 variant (501Y.V2), includes three different mutations (K417N, E484K, and N501Y) that do not interact with neutralizing antibodies, possibly leading to resistance to neutralization [102]. Several trials have been conducted to test the efficacy and safety of available vaccines, but it is still unclear how the different emerging strains are affecting vaccine efficacy [103]. The NVX-CoV2373 vaccine, for example, has been shown to be effective against both the Alpha and Beta variants [104, 105], and the BBV152/COVAXIN vaccine has also shown efficacy against the Alpha variant [106]. In contrast, several studies have shown decreased vaccine efficacy against some variants, including Alpha and Beta [103, 107–109].

The infamous Delta or B.1.167 variant, which first emerged in India in late 2020, contains a number of mutations at multiple sites, including D111D and E484R [110]. It has a significantly high transmissibility rate, with an R_0_ of almost 7, in comparison to 2.5 for the parental virus [101]. Moreover, a Scottish study found that infection with the Delta variant doubles the chance of hospitalization compared to infection with the Alpha variant [111]. Another study in Portugal showed that mRNA vaccines’ efficacy and impact on viral load was reduced against the Delta variant compared to the alpha variant [112]. While Public Health England confirmed that vaccines are indeed highly protective against symptomatic disease caused by the Delta variant, the efficacy was found to be only 33% after the first dose of either AstraZeneca or Pfizer-BioNTech vaccine [113]. Similarly, a 25-50% decrease in efficacy it was predicted for the Moderna and Oxford-AstraZeneca vaccines compared to the original virus [114]. More non-pharmaceutical interventions were also found to be required in order to curb the Delta variant sufficiently, even when vaccine coverage was high [115].

One of the variants that stands out for its high transmissibility is Omicron (B.1.1.529). This variant emerged late in November 2021 and showed the most genetic variation in the spike protein, with more than 30 substitutions, deletions, or insertions, which again raises concerns about the possibility of evading the immunity provided by vaccination [117]. In addition, this variant has a high transmission rate, which has led to rapid waves of infections globally [118, 119]. Fortunately, this variant’s clinical presentation was not as severe as that of the previous variants. Indeed, a study conducted on 43 patients infected with Omicron revealed that the most common clinical presentation was only flu-like (cough, congestion or rhinorrhea, and general fatigue), and only one of them needed hospitalization [117]. Importantly, no deaths were recorded amongst those Omicron-infected patients. Furthermore, the number of Omicron-infected patients who required oxygenation was significantly lower than with other variants [116]. Moreover, a study comparing Omicron to Delta showed a 65% and 83% decrease in the risk of hospitalization and intensive care unit admission or mortality, respectively [120]. Thus, this combination of high transmissibility and mild symptoms has allowed this variant to build herd immunity rapidly with relatively low morbidity and mortality rates, to the extent of even being referred to as “natural boosting immunity” [121]. This resulted in some European countries, including Denmark, to abruptly lift all social distancing restrictions, leading to a significant increase in the number of COVID-19 cases [122]. This illustrates the importance of building safe herd immunity in association with a gradual exit strategy.

While it is fortunate that mutated strains have so far not been shown to significantly affect vaccine efficacy [96], the continued mutation of SARS-CoV-2 raises doubts regarding their efficacy in the future and whether they will have to be modified continually [87, 97, 98]. Given the effects of these mutations on virus infection, spread, and immune resistance, it is possible that the efficacy of the vaccine may decline in people infected with those new strains [123]. In fact, some vaccine manufacturers are already preparing booster shots to provide protection against these variants [103]. The possibility that the COVID-19 vaccines may have to be re-evaluated and designed in case a strain evades protective immunity raises concerns about whether COVID-19 vaccines may eventually need to be changed or administered repeatedly, which might also impact the public’s trust and their compliance [124]. This is similar to how the vaccines against influenza are renewed annually to ensure efficacy against new strains [125]. Besides the main challenge mentioned above, there are other challenges, such as vaccine availability [16], misconceptions about the vaccine [19, 126], strict storage conditions that many countries may not be able to provide (especially third-world countries without the needed technology and resources) [127], and vaccine age restrictions [128].

New COVID-19 variants might arise in different countries due to geographic influences. For instance, the high genetic variability of SARS-CoV-2 has been found to be associated with climate differences in different geographic regions, leading to the development of more variant strains [94]. This raises the possibility that there may be more unidentified SARS-CoV-2 mutants that are currently present in different locations. In order to accommodate this significant genetic variability, vaccines containing genetic material from multiple SARS-CoV-2 variants might have to be developed, as is done with the influenza vaccine [129]. While it is fortunate that numerous COVID-19 vaccines are being developed, it is of essential importance to note that this does mean that they are accessible to everyone [15, 16].

In addition, there is mistrust of COVID-19 vaccines not only in the public but also among healthcare workers [130]. A study that surveyed hesitancy towards the COVID vaccines found that most countries had vaccine hesitancy levels higher than usual [19]. They concluded that to achieve a 67% level of herd immunity, vaccination programs will have to begin early and become mandatory. Moreover, they strongly recommend raising people’s awareness regarding COVID-19 vaccines and answer their questions and concerns at the social and national levels. In addition, younger populations will not be receiving vaccinations in most cases [131, 132]. Therefore, in the presence of all these vaccine challenges, governments are obligated to continue to implement effective control strategies to prevent COVID-19 transmission.

Since vaccinations have not been confirmed to prevent infection, the application of social distancing is still crucial in controlling the pandemic [15, 21]. Mathematical models have been used to predict the effect of the COVID-19 vaccine on virus transmission and social distancing. A predictive modeling study showed that vaccine coverage reaching up to 90% would lower virus transmission and, in turn, lower mortality rates [133]. To sufficiently control SARS-CoV-2 transmission, it has been calculated that a vaccine with at least 70% efficacy would have to be administered to two-thirds of the population, with the condition that non-pharmaceutical interventions (such as wearing masks and physically distancing) are not abruptly stopped [133]. Indeed, social distancing measures should not be stopped until at least eight months after at least 60% of the population has received a vaccine with 70% efficacy without waning immunity, or one with 90% efficacy with immunity waning after a year [133].

Similarly, a mathematical model predicting the impact of vaccine implementation in the US on COVID-19 transmission and mortality rate indicated that vaccination coverage of a minimum of 82% of the susceptible US population is needed to overcome the pandemic [134]. However, this percentage could be almost halved if everyone were to wear a mask. The model also illustrated that a 10% increase in vaccination coverage would lower the cumulative number of deaths from 120,000 to 56,000 [134]. Although the vaccine is predicted to have a significant effect, maintaining strict social distancing measures would cause an additional dramatic decrease in the cumulative mortality rate, reaching up to about 90%. Altogether, these results indicate the importance of the application of social distancing measures during vaccination, and both strategies should have a synergistic effect in eliminating the pandemic [29, 133, 134].

## Balanced social distancing and herd immunity during the COVID-19 pandemic

The uncertainties surrounding the rush to produce vaccines have affected the confidence of numerous individuals, and vaccine hesitancy has been growing [19, 126]. In addition, low-income governments around the globe may be uncertain of the next step to combat COVID-19 while struggling to get access to a vaccine. Therefore, it is important for each country to assess the advantages of keeping or lifting restrictive public health and social measures to be able to make critical decisions. Maintaining restrictive measures has been shown to reduce the risk of transmission, slow down the infection rate, and help in protecting high-risk individuals [26, 57, 61, 99, 135]. On the other hand, relaxing restrictive measures has helped some governments to improve their economy after the severe economic losses created by the pandemic, in addition to possibly helping to alleviate the mental stress and the impact of quarantine [136–139]. It is also important to note that lifting socially restrictive measures will aid in establishing herd immunity, which is essential for protection against another wave of SARS-CoV-2 infection, especially in countries with minimal access to the vaccine, or none at all.

Therefore, each government should propose a plan that finds a balance between the application of social distancing and establishment of herd immunity to maintain the critical control measures that ensure community safety. Gilbert and colleagues have suggested a strategy that reconciles the approaches of social distancing and herd immunity during COVID-19 infection, designed to carefully and gradually relax the strict physical distancing measures such as quarantine [140]. This strategy requires a significant increase in diagnostic capacity, which allows not only the infected but also the immune to be identified. Diagnostic tests target essential workers first, subsequently determining the risk profile of the workers. De-confinement starts with low-risk workers who have recovered, as well as immune individuals. Once COVID-19 is under control, recovered but non-immune individuals may be gradually de-confined as well, starting with younger individuals. Importantly, the authors stress the importance of maintaining preventative measures such as the isolation of the infected and testing their contacts, all while gradually de-confining the immunized.

## Balanced social distancing and herd immunity during vaccine implementation

For those countries that do not currently have a vaccine available or will not be able to implement a COVID-19 vaccination program within a year, a COVID-19 exit strategy has to be adapted. Rawaf and colleagues have proposed that a COVID-19 exit strategy plan should incorporate four public health principles: infection status, community acceptance, public health capacity, and health system spare capacity [141]. Governments should de-confine their populations in a stepwise manner, guided by these principles and infection indicators that are particular to the country and region. Although this plan could be based on ethical considerations, the decision of lifting restriction measures is multifaceted. Hence, moderate social distancing measures should be applied gradually in a structured and stepwise manner. This will ensure that virus transmission is strictly controlled to avoid a dramatic increase in the number of infections and prevent a collapse of the health system. Next, individuals must understand the importance of using masks, physical distancing, work shift hours, and avoiding crowded places, even if they are vaccinated. If they are not properly educated, and if social distancing measures are lifted, vaccinated individuals may not realize they can still be infected and spread the virus to others, even if their symptoms are mild or inapparent [142].

## Conclusion

Despite the development of vaccines, governments should ensure that they implement strategies to lower COVID-19 transmission rates. While social distancing has been one of the most common and effective strategies, it is unlikely that governments will be able to maintain such restrictive measures for a prolonged period due to their severe economic and social implications. Another – perhaps controversial – strategy is to promote natural herd immunity. Due to the ethical and health problems associated with allowing the virus to spread freely among the population, achieving herd immunity through natural infection alone is not a feasible solution in populations without equal and sufficient access to vaccines. Instead, we encourage a compromise strategy balancing social distancing and herd immunity. This balanced approach merges the benefits of both social distancing and herd immunity strategies, allowing the safe lifting of restrictive measures as herd immunity is gradually achieved, either by natural infection or vaccination. This strategy can be especially helpful now as vaccines have been developed and implemented in different countries to provide more protection to the population. Governments will be able to control travel measures in parallel with extensive testing, contact tracing, and quarantine following exposure. Thus, it is logical to assume that the implementation of these balanced measures would lead to a decline in SARS-CoV-2 transmission while aiding in the slow and safe buildup of herd immunity among different populations through natural infection and vaccination, if available. Importantly, it is crucial to raise the public’s awareness regarding vaccine concerns to avoid vaccine hesitancy and increase people’s trust in vaccines. Moreover, the public should be informed about why vaccines are not a green light to forgo protective measures.
